# Bright light therapy in the treatment of patients with bipolar disorder: A systematic review and meta-analysis

**DOI:** 10.1371/journal.pone.0232798

**Published:** 2020-05-21

**Authors:** Shengjun Wang, Zhigang Zhang, Li Yao, Nannan Ding, Lingjie Jiang, Yuchen Wu

**Affiliations:** 1 Intensive Care Unit, The First Hospital of Lanzhou University, Lanzhou, China; 2 School of Nursing, Lanzhou University, Lanzhou, China; Gachon University Gil Medical Center, REPUBLIC OF KOREA

## Abstract

The treatment of depressive symptoms of bipolar disorder (BD) has received increasing attention. Recently, some studies have shown that bright light therapy (BLT) seems to be useful for BD depression. This meta-analysis is intended to further elucidate the role of BLT in depressive symptoms in patients with BD. Register of Systematic Reviews PROSPERO: CRD 420191 33642.Randomized controlled trials and cohort studies were retrieved in PubMed, Cochrane Library, EMbase, Web of Science, CINHAL, CBM, CNKI, VIP, and Wanfang from their foundation to March 2020, and other sources as supplement was also retrieved. Data were extracted after strict evaluation of literature quality by two researchers, and Meta-analysis was conducted on literatures that met the inclusion criteria. Meta-analysis was performed using Revman 5.3 software. In total, 12 studies including 847 patients with BD depression were included in our meta-analysis. A meta-analysis found significant differences between BLT and placebo for the following outcomes: (1) depression severity before and after BLT [SMD = -0.43, 95% CI (-0.73,-0.13), P<0.05] in RCT and [SMD = -2.12, 95% CI (-2.3,-1.94), P<0.05] in cohort studies.; (2) the efficacy of duration/timing of light therapy for depressive symptoms in BD [I^2^ = 85%, SMD = -1.88, 95% CI (-2.04, -1.71),P<0.05] and [I^2^ = 71%, SMD = -2.1,95% CI(-2.24, -1.96), P<0.05]; (3) the efficacy of different color/color temperatures for depressive symptoms in BD [I^2^ = 0%, SMD = -0.56, 95% CI (-0.92, -0.19), P<0.05] and [I^2^ = 97%, SMD = -1.74, 95% CI (-1.99, -1.49), P<0.05].We performed a subgroup meta-analysis of studies that used different light intensities. The results showed that light intensity≥5000 lux significantly reduced the severity of depression. And patients without psychotropic drugs revealed significantly decreased disease severity [I^2^ = 0%, SMD = -0.6, 95% CI (-1.06,-0.13), P<0.05]. Limitations of the study include studies only assessed short-term effects, and insufficient duration may underestimate adverse reactions and efficacy. Our results highlight the significant efficiency of BLT in the treatment of bipolar depression. Prospective studies with more rigorous design and consistent follow-up.

## Introduction

Bipolar Disorder (BD) is a complex, chronic, sporadic mood disorder [1,2].It affects more than 1% of the world's population, and studies have shown that the total lifetime prevalence rates of BD I and II are 0.6%,and 0.4% respectively [3–6]. Formerly known as manic depression, BD is a serious chronic mood disorder characterized by manic episodes, hypomania and alternating or intertwined depressive episodes. Among these manifestation, depressive episodes are the most common, accounting for more than 50% of patients with BD [7,8]. BD is complicated, and the rates of misdiagnosis and missed diagnosis are high. The most widely acknowledged diagnostic classifications are the 10th revision of the International Classification of Diseases (ICD-10) and the 5th edition of the Diagnostic and Statistical Manual of Mental Disorders (DSM-5) [9–12]. Some studies have found that patients with BD make serious suicide attempts, and the annual mortality rate is higher than that of the general population [7,13–15]. Therefore the main purpose of treating depressive symptoms in BD is to quickly reduce disease severity and prevent suicide, which is considered the main challenge in long-term treatment [16–18]. Mood stabilizers and antipsychotics are the mainstays of acute treatment for mania and depression in BD [19], and lithium is considered to be one of the most effective treatments for preventing both types of symptoms [20]. However, given the high rate of recurrence of BD, these strategies can lead to complications such as nephrotoxicity or liver damage [21–23].

Bright light therapy (BLT), also called phototherapy, refers to the use of glare therapy to treat depressive symptoms [24], BLT was originally used to treat patients with seasonal affective disorder [25–29]. Numerous studies indicate direct and indirect effects of light on mood. One of the core symptoms of BD is difficulty sleeping [30–33], 20% of patients with BD also have sleep disorders, and circadian rhythm adjustment is a potential treatment. In circadian rhythm research, photoperiod was coded by the biological clock into the duration signal of nocturnal melatonin secretion, and melatonin secretion could be suppressed by bright light in humans. melatonin could be a mild soporific for many psychiatric sleep problems [34–36]. Wirz‐Justice et al. [34] combines information about clock gene variants, correlations with symptoms, neurotransmission and brain imaging found that serotonin (5-HT) neurotransmission, noradrenaline (NA),dopamine (DA) are all affected by sleep deprivation (SD). Multiple neurobiological effects can lead to clinical mood improvement. The conversion of neurotransmitters could provide a core biological underpinning for circadian preference in diurnal and for the antidepressant effects of chronotherapeutics. Garbazza et al. [37] reported multiple genetic mutations in clock mechanism linked to depression. Including gene polymorphisms of the core clock machinery or the seasonal change of daylight duration affects biological clock [34]. Cortical excitability normalizes the time course of its daily homeostatic variation. The circadian timing system and sleep homeostasis influence connectivity among brain areas, while functional connections between cerebral cortex areas is widely disrupted and is considered to be the main biological basis for emotional disorders and cognitive impairment [38]. Phototherapy is effective in treating depressive symptoms [39], and the sustained antidepressant effect of BLT has been confirmed in clinical studies [40,41]. In recent years, some systematic review and meta-analysis have investigated the effectiveness of BLT for patients with BD. some meta-analysis have suggested that BLT is effective [42–45], while others have shown that BLT does not have a significant antidepressant effect [46]. Lam et al. [42] reported that a small-to-moderate and significant effect of active light treatment in reducing depressive symptoms. But the evidence is positive but not conclusive. There are some limitations included different light treatment parameters, short treatment durations, small sample sizes and variable quality across trials. The International Society of Bipolar Disorders (ISBD) Task Force on Chronobiology and Chronotherapy [43] recommended BLT had the strongest evidence among current chronotherapeutic options. Limitations of this evidenced including the small number of RCTs and small sample sizes in each. Many different LT parameters were used and many of these patients were also taking mood stabilizing and other medications. Geoffroy et al. [44] shows a clear superiority of the combination of LT and AD. The main limitation is the small number of randomized trials which reduces statistical power and does neither allow further assessments of moderators nor to analyze all subgroups of patients. Tseng et al. [45] reported that treatment with BLT had statistically significant antidepressant effects. This study had some limitations included in the meta-analysis lacked a well-designed control group, the small number of RCTs and small sample sizes. However, another meta-analysis reported the opposite conclusion. Takeshima et al. [46] suggested BLT does not significantly improve depressive symptoms in BD. Importantly, no meta-analysis so far has examined the efficacy of duration / timing of light therapy and different color temperatures for depressive symptoms in BD.

In this systematic review and meta-analysis, we evaluated the efficacy of BLT for depressive symptoms in BD, and we determined the efficacy of duration / timing of light therapy and different color temperatures for depressive symptoms in BD, and we also evaluated subgroup analysis of auxiliary measures, effects of different light intensity / colors, and drugs on depressive symptoms. In addition, in order to expand the number of articles, we also searched 4 Chinese databases.

## Materials and methods

This meta-analysis was conducted based on the Preferred Reporting Items for Systematic Reviews and Meta-Analyses (PRISMA) Checklist and the Cochrane Handbook for Systematic Reviews (v5.1.0).

### Inclusion and exclusion criteria

The inclusion criteria were: (1) randomized controlled trial (RCT) or cohort study on the effects of BLT on depressive symptoms in patients with BD; (2) clearly defined diagnosis of BD. The exclusion criteria were: (1) data were incomplete or could not be extracted,(2) study subjects included pregnant women.

### Data sources and search strategies

A systematic literature search was performed within English (PubMed, Web of Science, Embase, Cochrane Library and CINHAL) and major Chinese(China National Knowledge Infrastructure, Wanfang, SinoMed and VIP) databases, from their inception dates to March,2020. The search was performed using the search terms “(bright light therapy OR light therapy OR light-therapy OR phototherapy OR light treatment OR Analytical, Diagnostic andTherapeutic Techniques and Equipment Category OR Therapeutics OR Color Therapy OR Heliotherapy OR Intense Pulsed Light Therapy OR Low-Level Light Therapy) AND (Psychiatry and Psychology Category OR Mental Disorders OR Bipolar and Related Disorders OR Bipolar Disorder OR Bipolar affective disorder)”. We used a combination of subject terms and free word retrieval and assessed related references. The search strategy are provided in S1 File. Two individuals simultaneously conducted independent searches according to the established inclusion and exclusion criteria, and EndNote X8 software was used for document management. In case of disagreement, a third person participated in the discussion until consensus was reached.

### Study selection

Using a standardized electronic form, two researchers independently completed the data extraction and checked. When a difference occurred, a third person participated in the discussion until consensus was reached. The extracted data include author names, publication year, sample size, intervention measures, country, research design, whether to use allocation concealment, blinding status, and outcome. The primary outcome was depression severity, as assessed by the Hamilton Depression Rating Scale (HDRS), Inventory of Depressive Symptomatology, Clinician Rating (IDS-C), or the Structured Interview Guide for the HDRS.

### Quality assessment

Two investigators independently evaluated the literature, Cohort studies and case-control studies were scored using the Newcastle Ottawa Scale (NOS) [47], and RCTs were evaluated using the Cochrane Handbook (5.1.0) bias risk assessment tool [48].

### Statistical analysis

Meta analysis was performed with RevMan 5.3 software. A meta-analysis aggregates indexes of effectiveness of individual trials into one pooled estimate. When the result is a continuous variable, then the effect size is usually expressed as mean difference (MD) or normalized mean difference (SMD).The outcome measure was analyzed using standardized mean differences (SMD). The MD is the difference in the means of the treatment group and the control group, while the SMD is the MD divided by the standard deviation (SD) [49]. Meta-analysis of trials that have used different continuous or rating scales to record outcomes of a similar nature requires sophisticated data handling and data transformation to a uniform scale, the standardized mean difference (SMD) [50].The measurement data adopt the standardized mean difference (SMD) as the effect index, and each effect quantity gives its point estimate and 95% confidence interval (CI). Heterogeneity among included studies was analyzed using χ2 tests (a = 0.1), and the magnitude was quantified in conjunction with I2. If there was no statistical heterogeneity between study results, a fixed effect model was used for meta-analysis; if there a random effect model was employed. Studies with obvious clinical heterogeneity were included in subgroup or sensitivity analysis, or only descriptive analysis [51]. In the first step, we conducted a separate meta-analysis of RCTs and cohort studies. To explore possible confounding effects of clinical variables, we also performed subgroup meta-analysis of studies based on different treatment parameters, including other auxiliary measures, light intensity and concomitant medication.

## Results

### Literature search results

**Fig 1:**
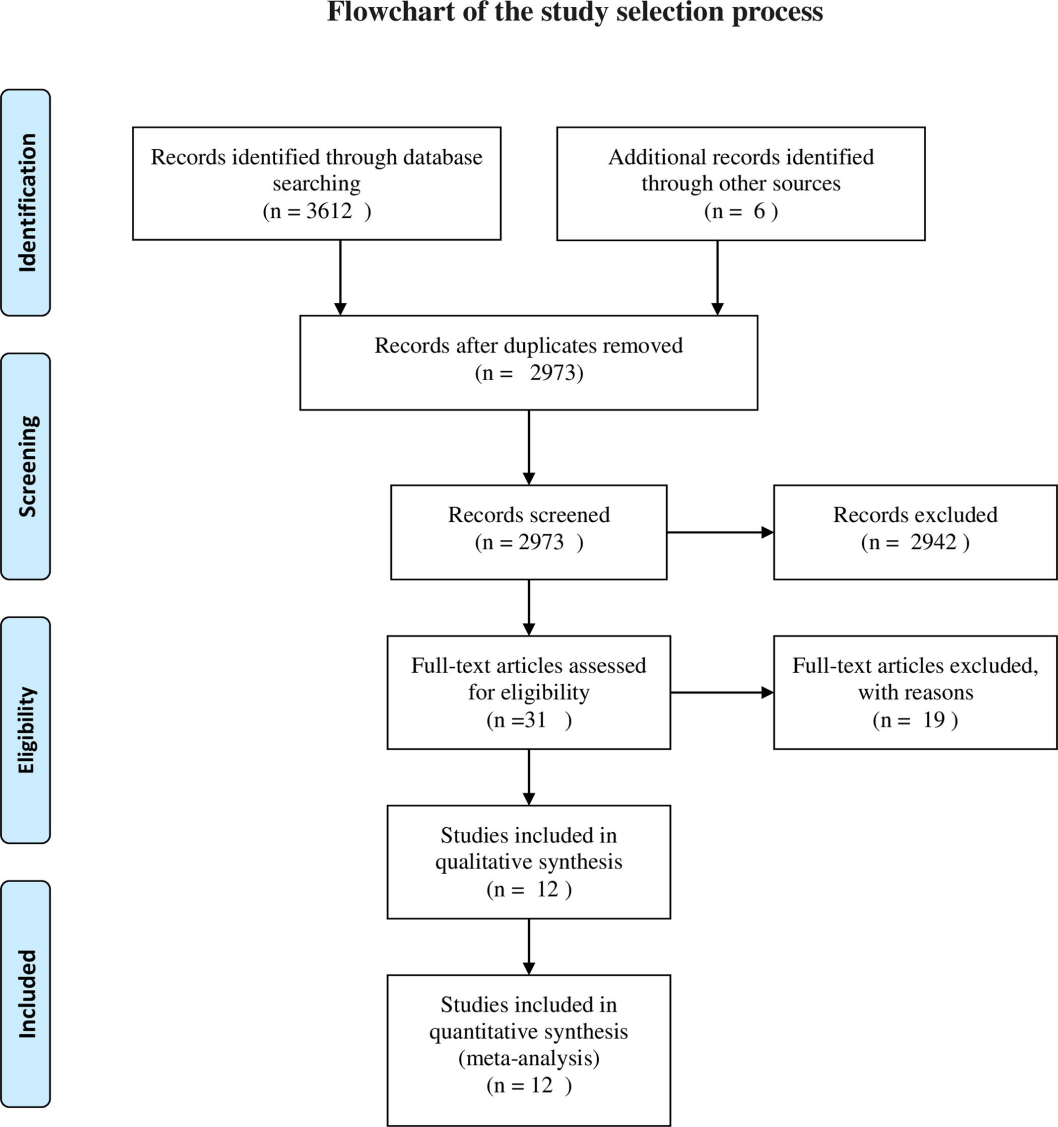
Study flow diagram.

A total of 3618 related publications were initially retrieved. According to the inclusion and exclusion criteria, 12 were selected after screening [52–63]. The study flow diagram is shown in Fig 1.

### Study and patient characteristics

The include studies are described in Table 1. A total of 12 articles including 5 RCTs and 7 cohort studies were include with samples of 7220 patients for a total of 847 cases. The research publication period was from 1995 to 2018, and 7 articles were published after 2010. The DSM-IV criteria were used for BD diagnosis in all studies. With regard to literature quality, 4 RCTs were grades B and 1 was C. Among cohort studies, 1 study scored 8 points on the NOS, 4 had 7 points, 1 had 6 points, and 1had 5 points.

**Table 1:** Characteristics and quality assessment result of the included studies.

Study	Country	Numbers	Diagno-stic criteria	Subjects	Mean age (year)	Intervention duration(h)	Color Temperature(k)	Timing	Light intensity (lux)	Light color	Drug free	Study type	Quality
Zhou2018	China	37/37	DSM-IV	BD, depress	35.1±14.2	14	4000	morning	5000	White	Yes	RCT	B
Sit D2018	USA	23/23	DSM-IV	BD, depress	45.7±14.3	31.5	10000	midday	7000	White	No	RCT	B
D.Sikkens2018	Holland	10/10	DSM-IV	BD, depress	47.6±16.9	5	N\A	morning	10000	White	No	Cohort study	5
Suzuki2018	Italy	220/220	DSM-IV	BD-I, depress	46.8±11.2	5	4600	morning + night	10000	White	No	Cohort study	7
Suzuki2016	Italy	147/147	DSM-IV	BD-I, depress	47.4±10.8	8.5	4600	morning + night	10000	White	No	Cohort study	7
Benedetti2014	Italy	143/143	DSM-IV	BD-I, depress	47.2±11.6	8.5	4000	morning + night	10000	White	Yes	Cohort study	8
Dauphinais2012	USA	18/20	DSM-IV	BD, depress	42.4±12.4	36.3	N\A	morning	7000	N\A	No	RCT	B
Wu2009	USA	32/17	DSM-IV	BD, depress	39.4±13.6	6	N\A	morning	5000	N\A	Yes	RCT	C
Benedetti 2009	Italy	44/44	DSM-IV	BD-I, depress	46.6±9.5	3	N\A	morning + night	400	Green	Yes	Cohort study	7
Benedetti 2007	Italy	39/39	DSM-IV	BD-I, depress	45.5±13.2	3	N\A	morning + night	400	Green	Yes	Cohort study	7
Benedetti 2003	Italy	18/12	DSM-IV	BD, depress	54.3±11.3	7	N\A	morning	400	Green	No	RCT	B
Papatheodorou 1995	Canada	7/7	DSM-IV	BD, depress	19.4±2.0	10.5	N\A	morning + night	10000	White	No	Cohort study	6

Abbreviation: BD-I: bipolar I disorder; BD: bipolar disorder;; N\A: not available; DSM-IV: Diagnostic and Statistical Manual of Mental Disorders, 4th Edition; Data presentation: mean ±SD

### Main meta-analysis results

The primary outcome measures included the following: (1) depression severity before and after BLT; (2) the efficacy of duration / timing of light therapy for depressive symptoms in BD; (3) the efficacy of different color/color temperatures for depressive symptoms in BD.

**Fig 2:**
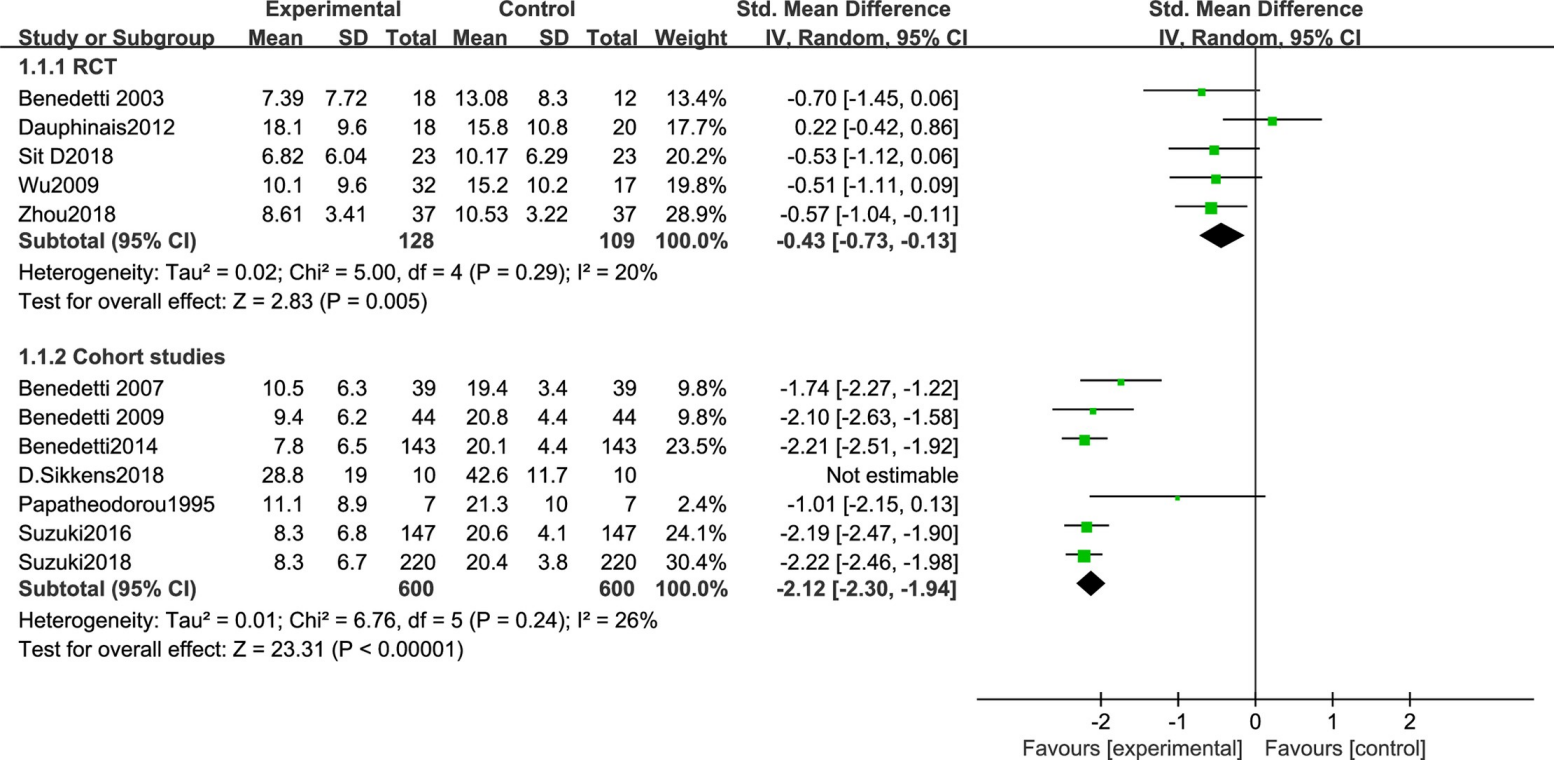
Meta-analysis results of depression severity in RCT group and cohort study group.

We first analyzed studies comparing depressive severity before and after BLT. A total of 12 articles [44–55] were included. Because of the different research designs, the cohort studies and RCTs were analyzed separately. There was less heterogeneity between RCTs [44,45,50,51,54] (I^2^ = 20%, P = 0.29), so a random effects model was employed. The results showed that BLT significantly reduce depression severity [SMD = -0.43, 95% CI (-0.73,-0.13), P<0.05]. A total of 7 cohort studies were included [46–49,52,53,55], and a sensitivity analysis was conducted because of the high heterogeneity. This decreased after omitting Sikkens et al. (I^2^ = 26%, P = 0.24). The findings revealed that BLT significantly decrease the severity of depression [SMD = -2.12, 95% CI (-2.3,-1.94), P<0.05] (Fig 2). Disease severityas was significantly lower in the patients with BD-D episodes after light therapy, either in the form of monotherapy or in combination with other treatment (sleep deprivation or lithium therapy).

**Fig 3:**
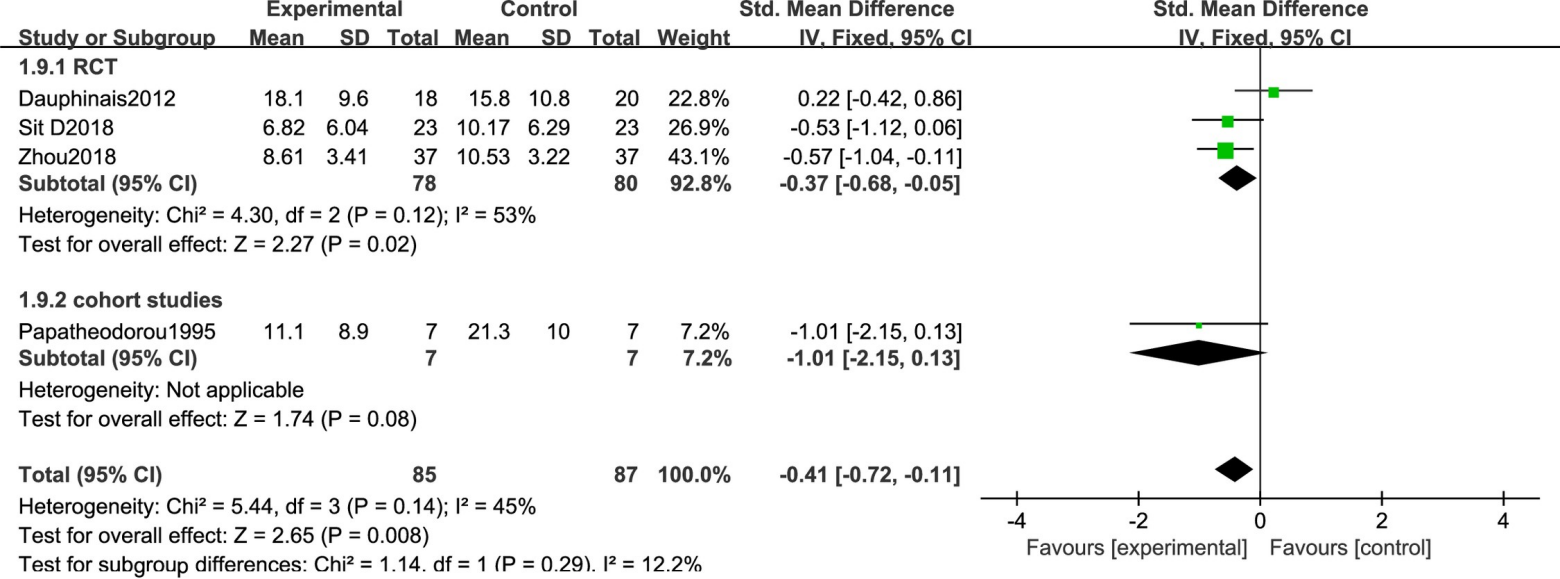
Forest plots with phototherapy greater than 10h.

**Fig 4:**
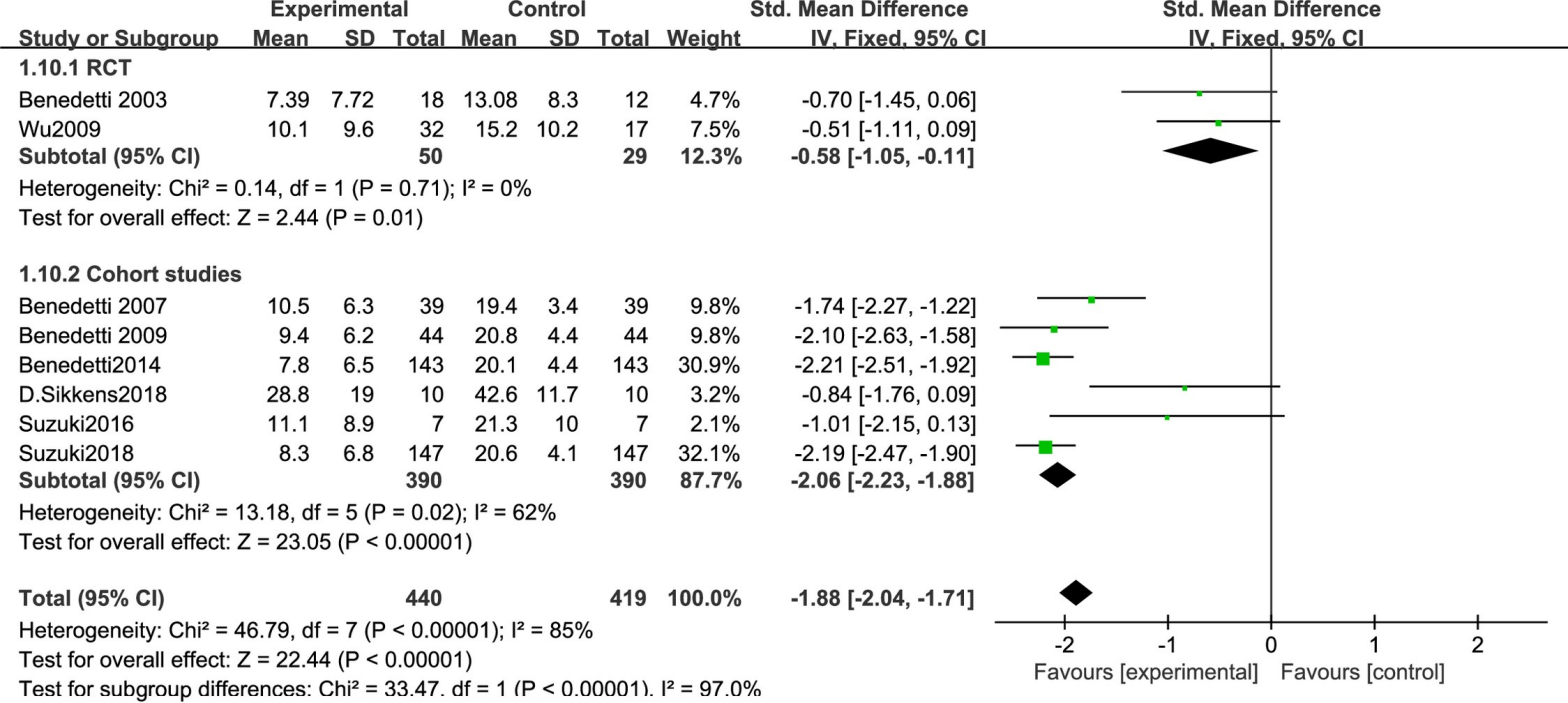
Forest plots with phototherapy less than 10h.

**Fig 5:**
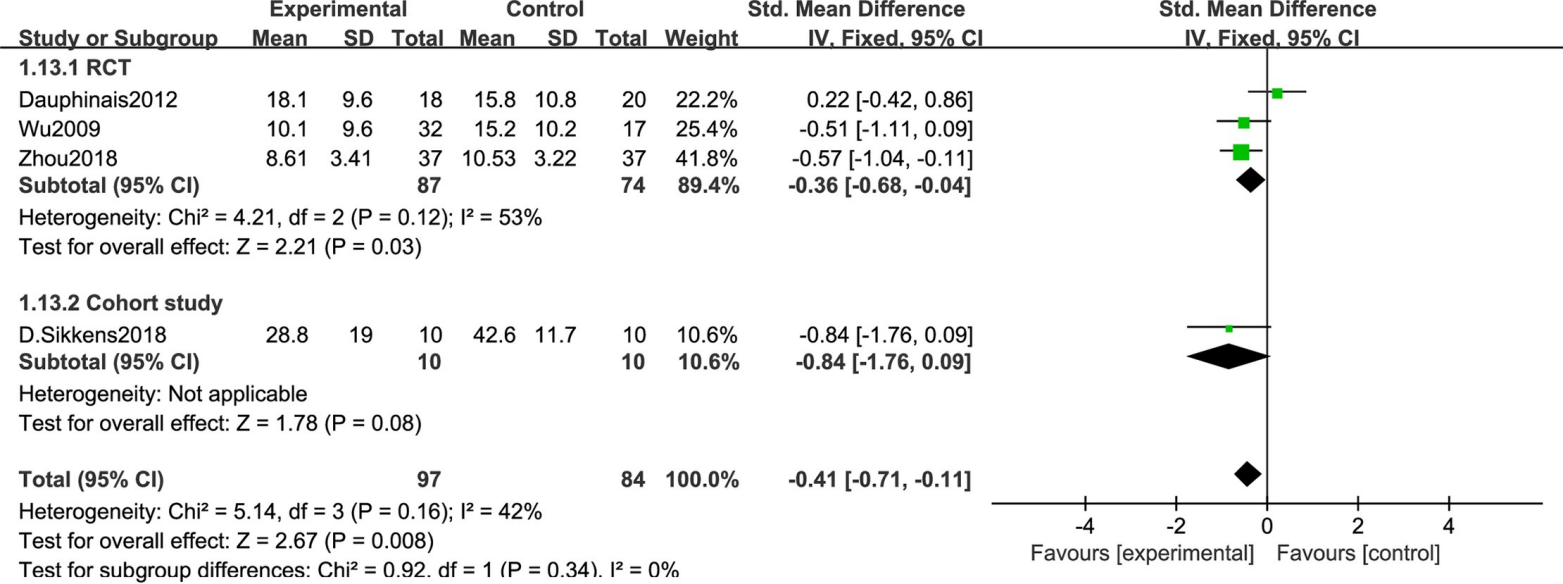
Forest plots for morning phototherapy.

**Fig 6:**
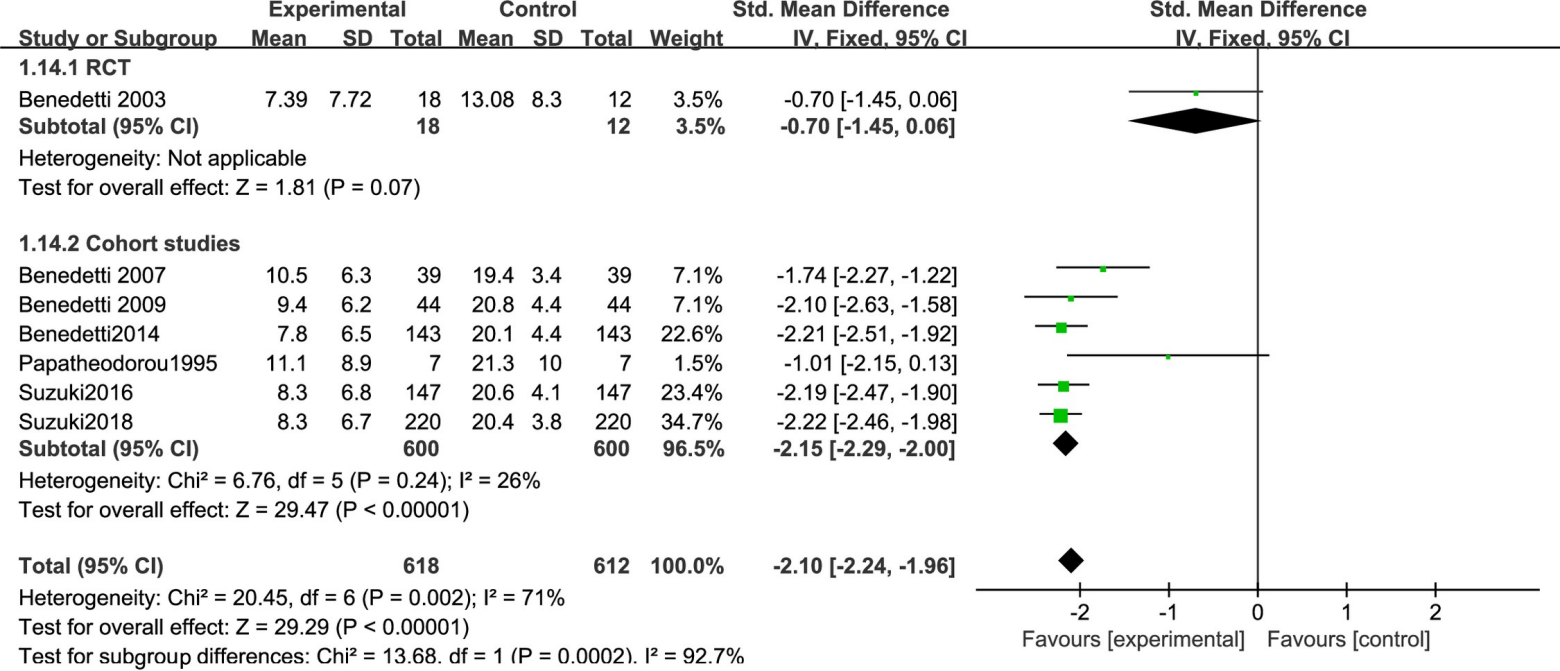
Forest plots for morning plus night phototherapy.

Fig 3 shows the efficacy of duration of light therapy for depressive symptoms in BD. There were 4 studies with phototherapy greater than 10 hours, including 3 RCTs [50,51,56] and 1 cohort study [61] [I^2^ = 45%, SMD = -0.41, 95% CI(-0.72, -0.11),P<0.05]; less than 10h included 2 RCTs [57,60] and 6 cohort studies [52–55,58,59] [I^2^ = 85%, SMD = -1.88, 95% CI(-2.04, -1.71),P<0.05] (Fig 4). Light conditions were superior to light control conditions in clinician-rated depressive symptoms. Fig 5 shows the efficacy of timing of light therapy for depressive symptoms in BD. We analyzed the effects of different phototherapy timings on depression. 4 articles [50,52,56,57] used morning light therapy [I^2^ = 42%, SMD = -0.41, 95% CI(-0.71, -0.11),P<0.05] and 7 articles [53–55,58–61] used morning plus evening light therapy[I^2^ = 71%, SMD = -2.1, 95% CI(-2.24, -1.96),P<0.05] (Fig 6), the analysis showed significant difference between conditions.

**Fig 7:**
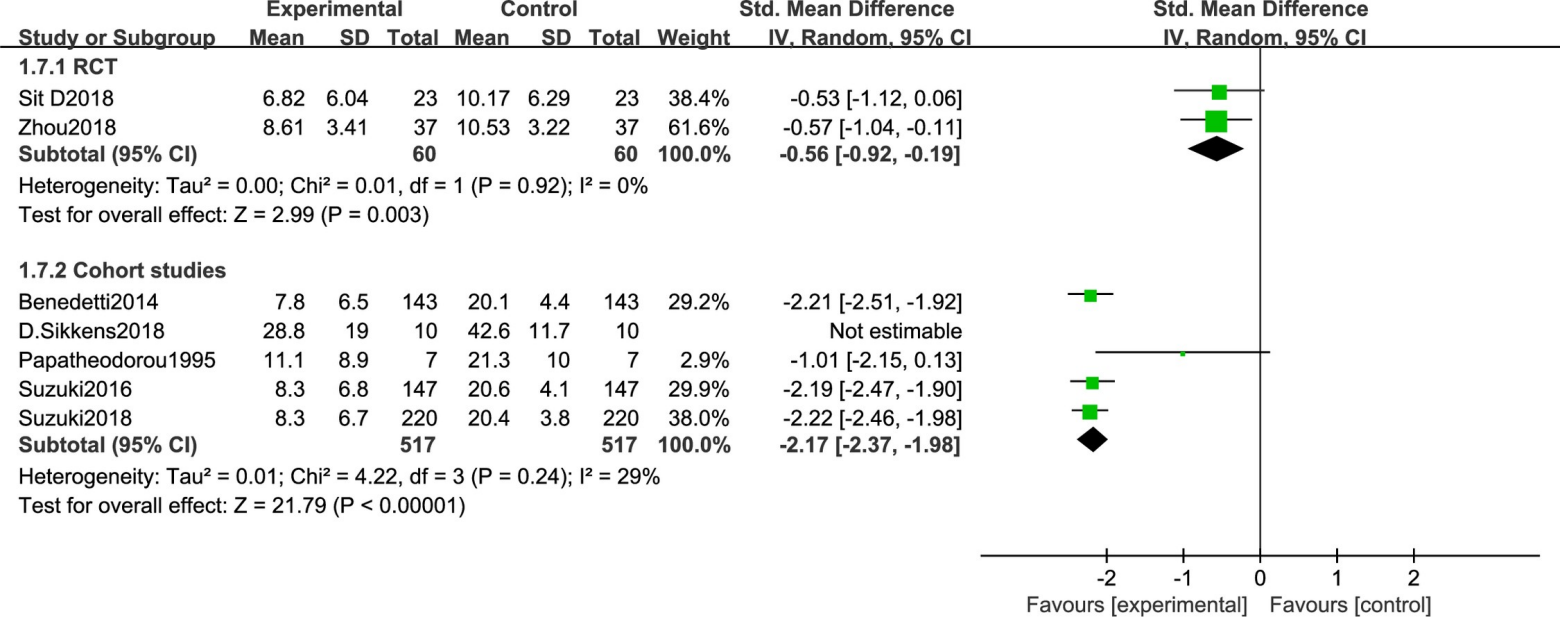
Forest plots for white light therapy.

**Fig 8:**
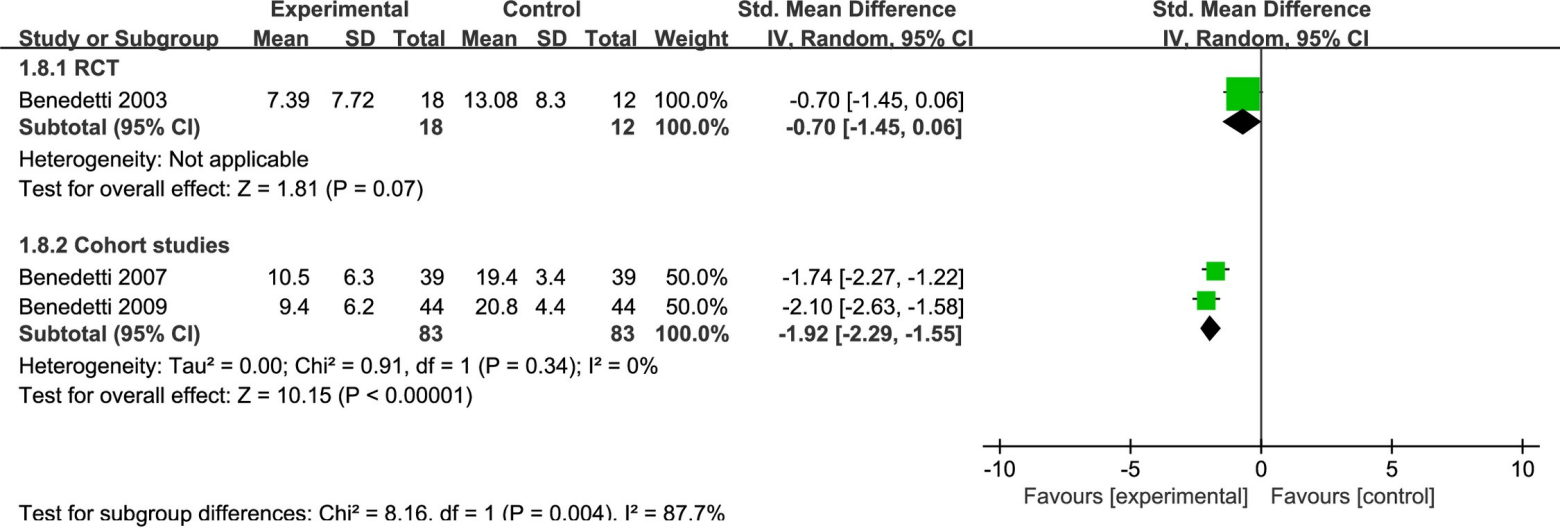
Forest plots for green light therapy.

**Fig 9:**
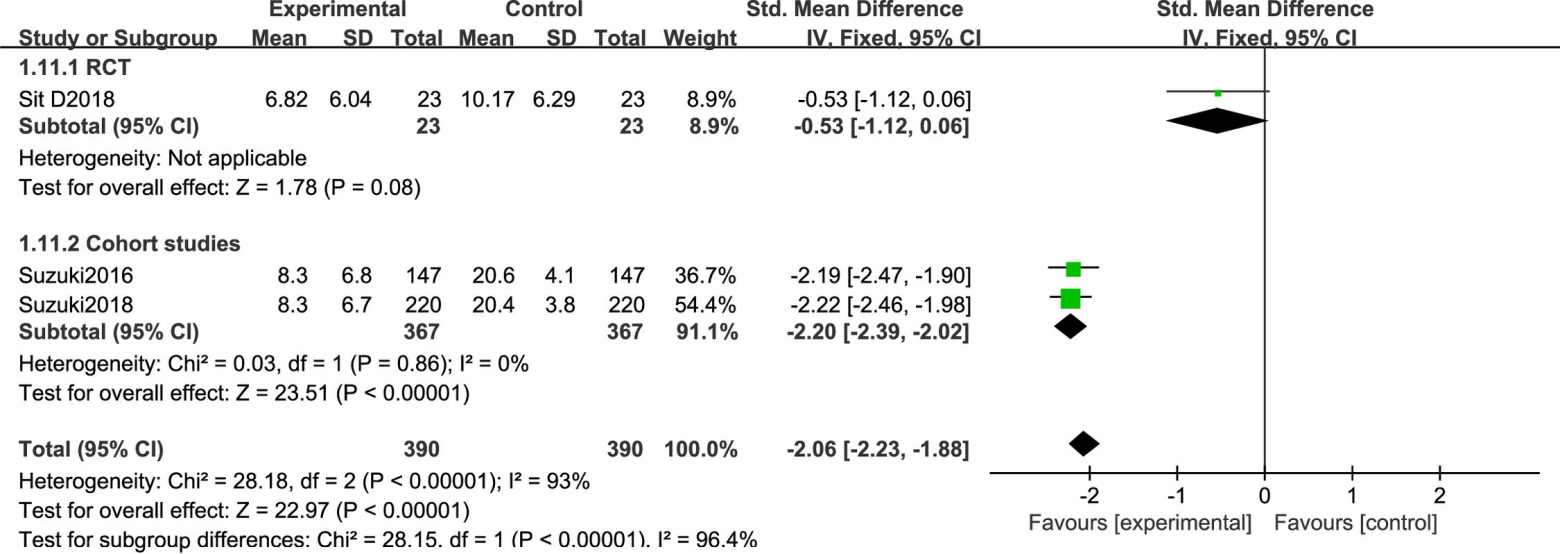
Forest plots with a color temperature greater than 4500k.

**Fig 10:**
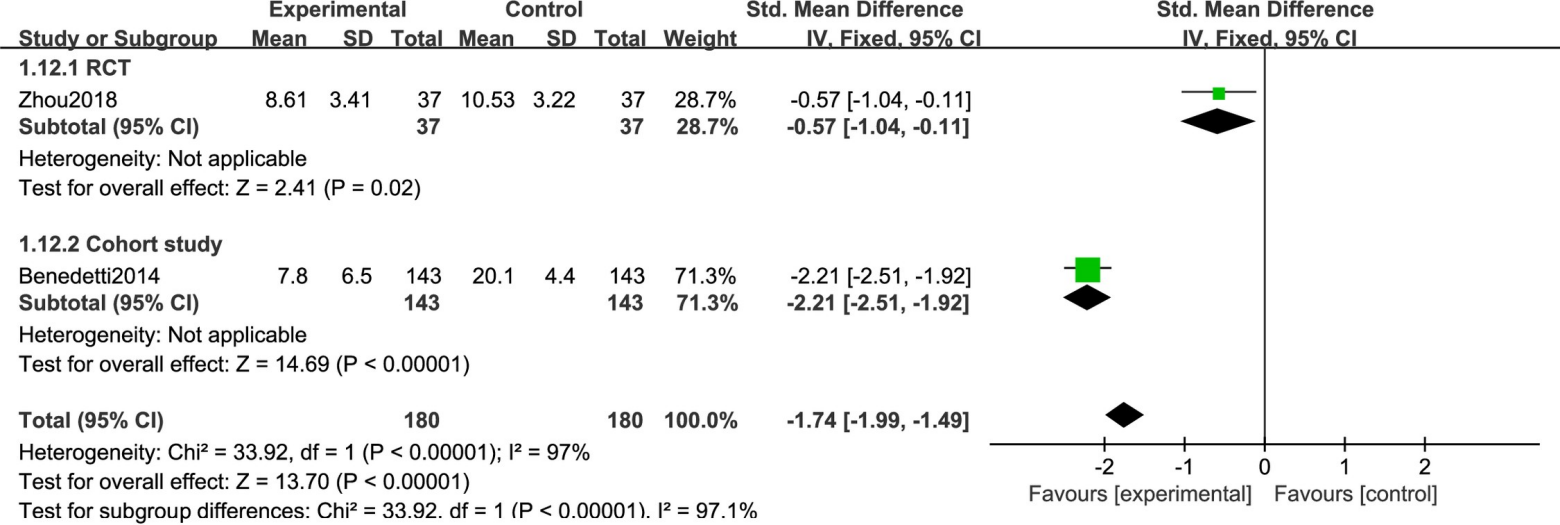
Forest plots with a color temperature less than 4500k.

Different light color were used in the included study, and the effect of different light color on the treatment of patients is inconclusive. Therefore, we performed a meta-analysis of studies that used different light color. 7 articles were include: 2RCTs [50,51] and 5 cohort studies [53–56,61], that were discussed separately according to the study design. We found that white light therapy resulted in significantly decreased disease severity in BD patients, including RCT [I^2^ = 0%, SMD = -0.56, 95% CI (-0.92, -0.19), P<0.05] (Fig 7), cohort studies [I^2^ = 29%, SMD = -2.17, 95% CI (-2.37, -1.98), P<0.05]. Because of the high heterogeneity, we conducted a sensitivity analysis. Three studies used green light. We found that in one RCT study [60], P was greater than 0.05,which was not statistically significant[SMD = -0.7,95%CI(-1.45, 0.06), P>0.05],while the other two cohort studies [58,59] showed green light therapy resulted in significantly decreased disease severity in BD patients[I^2^ = 0%, SMD = -1.92, 95% CI(-2.29, -1.55),P<0.05] (Fig 8).Data for color temperatures were available for five trials. There were three studies with color temperatures greater than 4500k, and two studies with less than 4500k. The studies of color temperatures greater than 4500k showed light superior to control conditions for improvement in depressive symptoms[I^2^ = 93%, SMD = -2.06, 95% CI(-2.23, -1.88),P<0.05] (Fig 9). Similarly, the studies of color temperatures less than 4500k showed differences between light and control conditions in improvement in depressive symptoms[I^2^ = 97%, SMD = -1.74, 95% CI(-1.99, -1.49),P<0.05] (Fig 10).

### Subgroup meta-analysis results of studies with or without auxiliary measures

**Fig 11:**
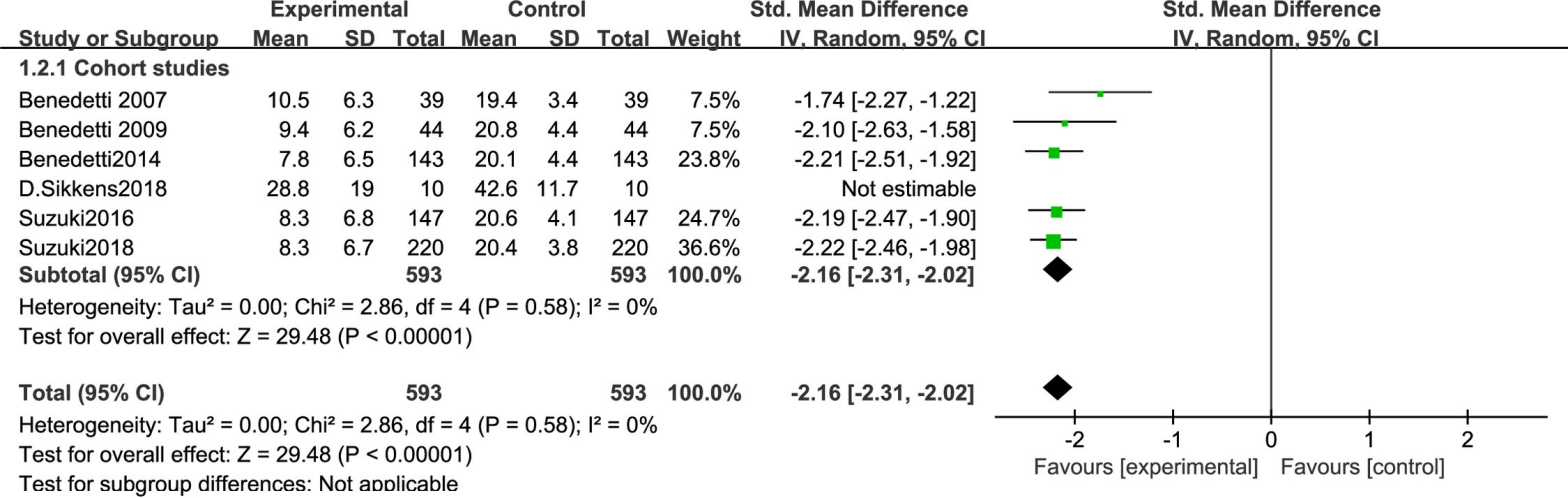
Forest plots with auxiliary measures.

**Fig 12:**
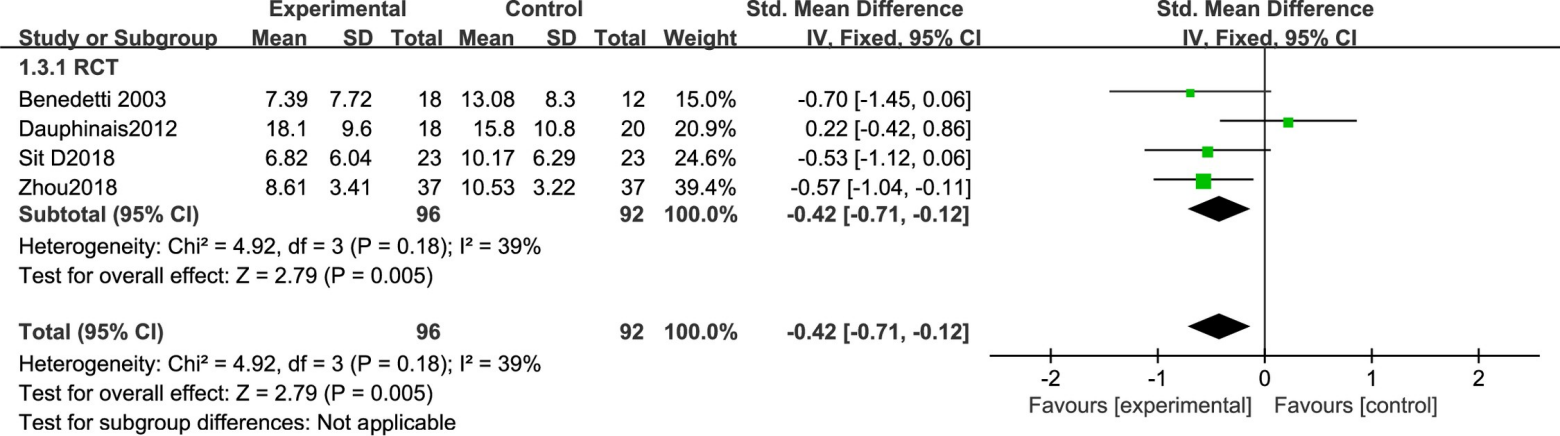
Forest plots without auxiliary measures.

To exclude a possible confounding effect of auxiliary measures, we performed a subgroup meta-analysis. Auxiliary measures mean that in some studies, researchers used sleep deprivation or lithium therapy to treat BD.A total of 7 articles used auxiliary measures: 6 cohort studies [46–49,52–53] and 1 RCT [51]. Since the number of RCTs was too small, we only assessed the 6 cohort studies. A sensitivity analysis showed significantly decreased depression severity in BD patients after BLT with auxiliary measures [I^2^ = 0%, SMD = -2.16, 95% CI (-2.31,-2.12, P<0.05] (Fig 11).Five articles did not use auxiliary measures, 4 RCTs and 1 cohort study. Analysis of the RCTs revealed significantly decreased depression severity in BD patients after BLT with auxiliary measures [I^2^ = 39%, SMD = -0.42, 95% CI (-0.71,-0.12, P<0.05] (Fig 12).

### Subgroup meta-analysis result of studies with different light intensities

**Fig 13:**
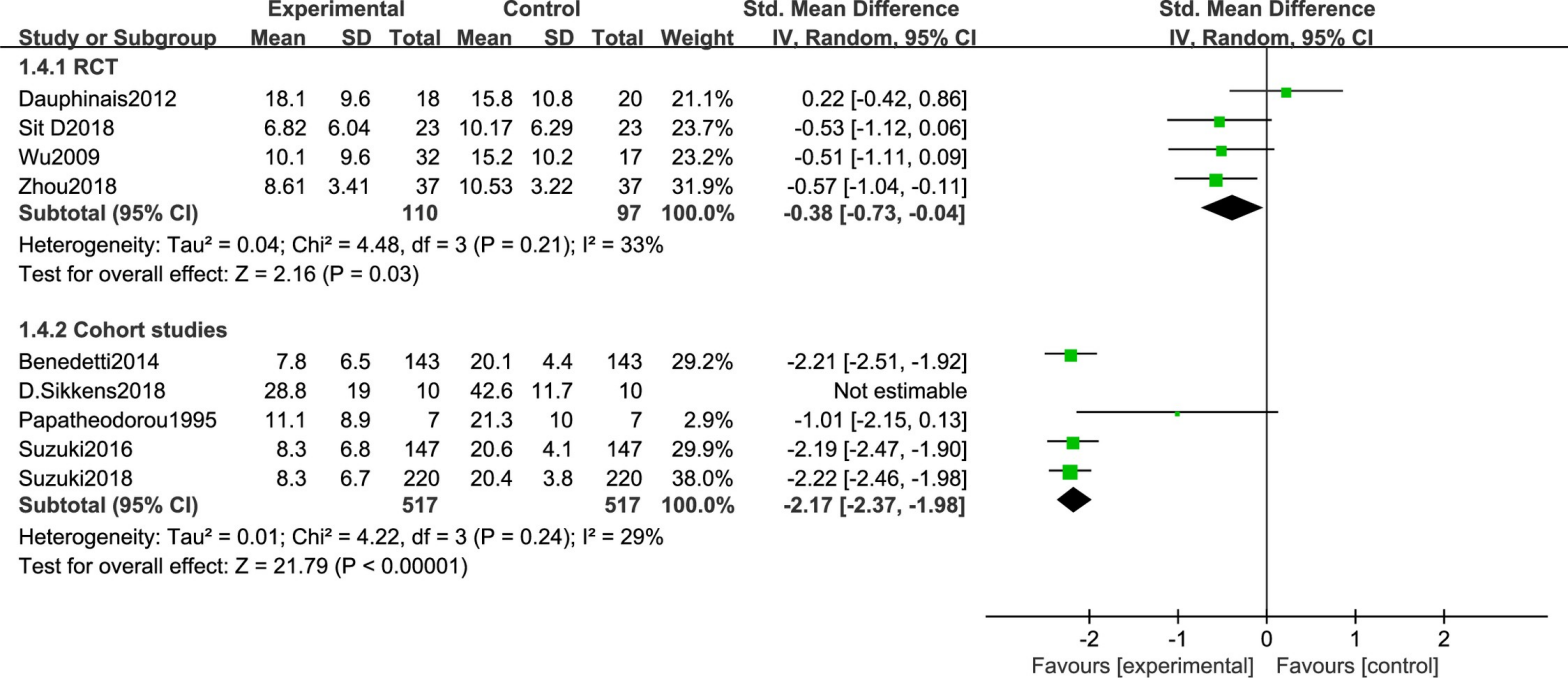
Forest plots with light intensities greater than 5000lux.

**Fig 14:**
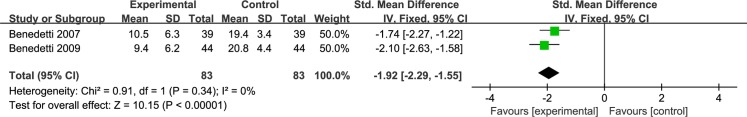
Forest plots with light intensities less than 5000lux.

We performed a subgroup meta-analysis of studies that used different light intensities. Nine articles were include: 4 RCTs [44–45, 50–51] and 5 cohort studies [46–49, 55], that were discussed separately according to the study design. The meta-analysis of the RCTs showed that: light intensity ≥5000 lux significantly reduced the severity of depression [I^2^ = 33%, SMD = -0.38, 95% CI (-0.73,-0.04), P<0.05]. Because of the high heterogeneity among the cohort studies (I^2^ = 67%, P = 0.02), we conducted a sensitivity analysis. Heterogeneity decreased after omitting Sikkens et al. (I^2^ = 29%, P = 0.24), and the subgroup meta-analysis of cohort studies revealed that light intensity ≥ 5000 lux significantly reduced depression severity [I^2^ = 29%, SMD = -2.17, 95% CI(-2.37,-1.98), P<0.05] (Fig 13). There were 3 studies with light intensities≤5000 lux,2 cohort studies [52,53], and 1 RCT [54]. Only the cohort study results were assessed. We found that even low light intensities could reduce the severity of depression [I^2^ = 0%, SMD = -1.92, 95% CI (-2.29,-1.55), P<0.05] (Fig 14).

### Subgroup meta-analysis results of studies without psychotropic drugs

**Fig 15:**
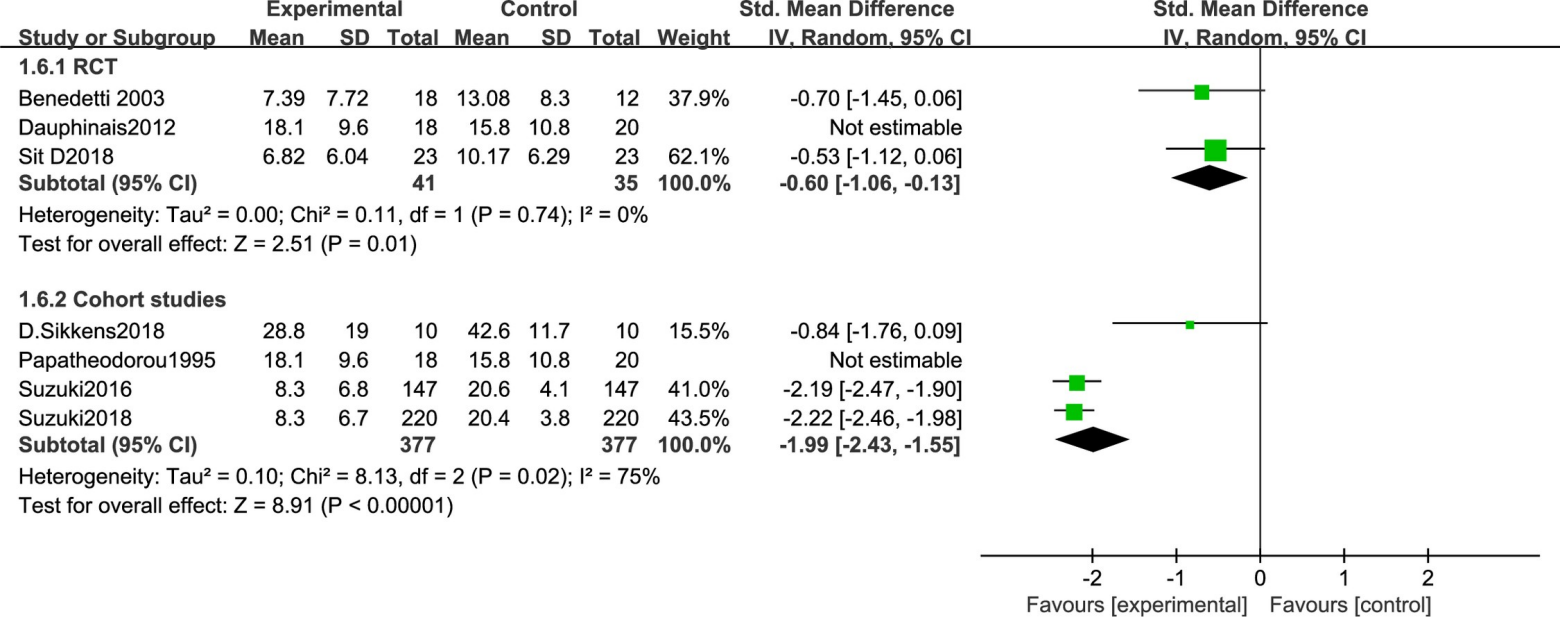
Forest plots with without psychotropic drugs.

To rule out possible confounding effects of drug treatment, we performed a subgroup meta-analysis focusing on BLT in the absence of any psychotropic drug prescriptions. Seven articles [45–48, 50, 54–55] were included. Because of the different research designs, the cohort studies [46–48,55] and RCTs [45,50,54] were analyzed separately. Sensitivity analysis, revealed significantly decreased disease severity in BD patients after BLT without psychotropic drugs in RCTs [I^2^ = 0%, SMD = -0.6, 95% CI (-1.06,-0.13), P<0.05] (Fig 15)and cohort studies [I^2^ = 75%, SMD = -1.99, 95% CI (-2.43,-1.55), P<0.05] (Fig 15). Given the small number of studies and high heterogeneity, this finding requires confirmation.

## Discussion

According to the published 2010 Global Burden of Disease Study, BD is the 18th most disabling health condition in the world and the treatment of depression in this population is a major challenge, with few effective approaches [64–67]. We also found that the efficacy of antidepressants for BD requires more evidence [68–70]. Besides pharmacologic treatment, studies have assessed the use of cognitive-behavioral therapy, social rhythm therapy, and family-centered treatment [32,64,71,72]. BLT is recognized as an alternative to psychotherapy and psychopharmacology for treating adults with depression [73], and there is ample evidence that morning BLT is effective and safe for depression in BD [74–77]. A study of phototherapy for bipolar disorder also showed that phototherapy has a treatment for bipolar disorder [45]. However, due to the different types of research design and the quality of the literature, Tseng’s has different quality and limitations. Therefore, our study will discuss the randomized control group and the cohort study group separately, taking into account the confounding factors such as color, intensity and drug impact.

To our knowledge, this is the first systematic review and meta-analysis that has evaluated the effectiveness of BLT in different colors, color temperatures, and duration / time for depressive symptoms in BD. The main results were that depressive severity decreased after BLT, and that treatment effect were observed, with different light color / color temperatures, with different duration / time, with or without auxiliary measures, with different light intensities, and without psychotropic drugs. The first result confirms that both RCTs and cohort studies showed that BLT can reduce depressive severity, indicating that it is feasible and effective in patients with BD. The mechanism by which BLT ameliorates depressive symptoms is likely accomplished by adjusting sleep-wake rhythms, inhibiting melatonin secretion, and increasing serotonin and norepinephrine levels [78,79].

Subgroup analyses were performed to eliminate the effects of auxiliary measures, light intensity, and psychotropic drug. In a subgroup analysis of adjuvant therapy, we found that phototherapy was effective in improving the degree of depression with or without adjuvant therapy. In the subgroup analysis of light intensity, the result was statistically significant, but only 2 studies used a light intensity≤ 5000 lux, so confirmatory results are required. In a subgroup analysis for drug interventions, significant effects for BLT were found regardless of pharmacologic treatment. In addition, our subgroup analysis revealed that the study by Sikkens et al. (2018) introduced significant, heterogeneity that almost disappeared after its omission. This may be the study did not report the basic subject characteristics in detail, and the document quality score was only 5 points. In addition, the IDS-C scale was used as the outcome, which is different from the other studies and may have introduced bias. In this meta-analysis, only 2 studies [44,45] applied BLT as the sole intervention, underscoring the need for more large-sample RCTs.

### Limitation

This major limitation of this meta-analysis is that the included studies only assessed short-term effects, and insufficient duration may underestimate adverse reactions and efficacy. Rigorously designed RCTs are needed to clarify the benefits and adverse effects of BLT for depression in BD.

### Conclusion

In summary, Our results highlight the significant efficiency of BLT in the treatment of bipolar depression, and we determined the efficacy of duration / timing of light therapy and different color temperatures for depressive symptoms in BD, and we also evaluated subgroup analysis of auxiliary measures, effects of different light intensity / colors.However, the existing evidence is not sufficient, and there are no uniform standards for light box use or light intensity standards. Prospective studies with more rigorous design and consistent follow-up periods are needed to confirm the effects of BLT on depressive symptoms in patients with BD.

## Supporting information

S1 File PubMed search strategy. (DOCX)

S2 File PRISMA 2009 checklist. (DOC)
