# Supplementary material for: Bright light therapy in the treatment of patients with bipolar disorder: A systematic review and meta-analysis
Source: PLoS One. 2020 May 21;15(5):e0232798. doi: 10.1371/journal.pone.0232798 (PMC7241702; doi:10.1371/journal.pone.0232798)
Supplement: S1 File — (DOCX) [file pone.0232798.s001.docx]

**S1:**

**Search strategies**

Pubmed:

1. Search "Bipolar Disorder"[Mesh]
2. Search((((((Psychiatry[Title/Abstract] AND Psychology Category [Title/ Abstract])) OR Mental Disorder[Title/Abstract] OR (Bipolar [Title /Abstract] AND Related Disorders [Title/Abstract] )) OR Bipolar Disorder[Title/Abstract]) OR bipolar depression [Title/Abstract]) OR Bipolar affective disorder [Title/Abstract]
3. Search 1 OR 2
4. Search "Phototherapy"[Mesh]
5. Search(((((((((bright light therapy [Title/Abstract]) OR light therapy [Title/Abstract]) OR phototherapy [Title/Abstract]) OR light treatment [Title/Abstract]) OR (Analytical, Diagnostic [Title/Abstract] AND Therapeutics [Title/Abstract]) OR Color Therapy [Title/Abstract]) OR Heliotherapy [Title/Abstract]) OR Intense Pulsed Light Therapy [Title/Abstract]) OR Low-Level Light Therapy [Title/Abstract]
6. Search 4 OR 5
7. Search 3 AND 6
